# *Chlamydophila psittaci* in Fulmars, the Faroe Islands

**DOI:** 10.3201/eid1202.050404

**Published:** 2006-02

**Authors:** Björn Herrmann, Heléna Persson, Jens-Kjeld Jensen, Høgni Debes Joensen, Markus Klint, Björn Olsen

**Affiliations:** * University Hospital, Uppsala, Sweden;; † Kalmar Hospital, Kalmar, Sweden;; ‡ The Faroese Museum of Natural History, Tórshavn, Faroe Islands;; § Office of Chief Medical Officer, Tórshavn, Faroe Islands;; ¶ Umeå University, Umeå, Sweden;; # University of Kalmar, Kalmar, Sweden

**Keywords:** Chlamydophila psittaci, fulmar, psittacosis, avian chlamydiosis, chlamydophilosis, the Faroe Islands, dispatch

## Abstract

*Chlamydophila psittaci* was detected in 10% of 431 fulmars examined from the Faroe Islands. Analysis of *ompA* showed a sequence almost identical to that of the type strain. The origin of *C. psittaci* outbreaks in fulmars is discussed. Despite a high level of exposure, the risk for transmission of *C. psittaci* to humans is low.

During the winter of 1929–1930, widespread epidemics of chlamydophilosis (psittacosis) occurred in Europe and the United States, and the causative agent was isolated from humans and affected birds (*1*). Presumably the epidemics originated in Argentina (*2*), and the disease was exported by shipments of pet birds. From the Faroe Islands (Figure), 174 cases of human chlamydophilosis were reported from 1930 to 1938 (*3*). The human death rate was 20%; it was especially high (80%) in pregnant women. Experimental work confirmed that "psittacosis virus" was contracted by humans when juvenile fulmars (*Fulmarus glacialis*) were caught and prepared for cooking (*4*). After the outbreaks in the 1930s, hunting fulmars for human consumption was prohibited until 1954, and data on chlamydophilosis have been scarce. In this study, our aim was to determine the current prevalence of *Chlamydophila psittaci* in fulmars and to relate it to available information on chlamydophilosis in humans in the Faroes.

**Figure:**
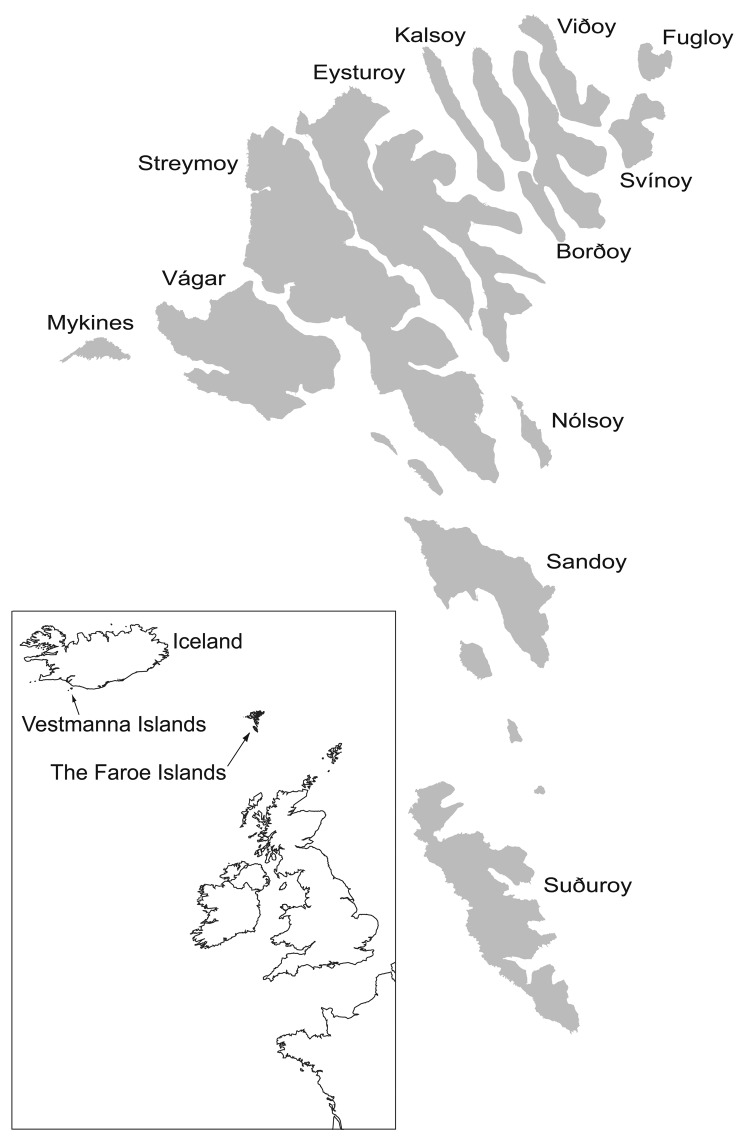
Map of the northern Atlantic showing the Faroe Islands and surrounding areas. Source: Faroese Museum of Natural History. Adapted by Janus Hansen.

## The Study

Cloacal swab samples were collected in September 1999 from 431 nonflying, juvenile fulmars. Samples were stored in sucrose–phosphate-buffered saline (PBS) in dry ice and transported to Sweden for analysis.

DNA was extracted from 400 μL of each sample (High Pure PCR Template Preparation, Roche, Branchburg, NJ, USA) and eluted in 100 μL of buffer. *C. psittaci* was detected by using 23S rRNA-based quantitative polymerase chain reaction (PCR) (*5*). A positive control was used for each round of DNA extraction and PCR. To monitor contamination, 1 negative control of PBS was included for every 5 samples in each run of DNA extraction and the ensuing PCR.

To characterize *Chlamydophila* cases, a 1,101-bp PCR fragment of *ompA* was sequenced. A nested PCR was used; the outer primers were FOMPF1 5´-GAAATCGGCATTATTRTTTGCC-3´ and FOMPR2 5´-CCAGTGATTGACCATTTGTCA-3´. Initial amplification was performed by using 0.2 μmol/L of each primer, 200 μmol/L deoxynucleoside triphosphates, 2 U Taq DNA polymerase (Qiagen, Hilden, Germany) in 1.5 mmol/L MgCl_2_ and 5 μL template DNA. Thermal conditions included 40 cycles of 20 s at 95°C, 60 s at 52°C, and 80 s at 72°C. In a second amplification, the primers FOMPF2 5´-TACGGGTTCCGCTCTCTC-3´ and FOMPR1 5´-CATTTGTCAGCGTCGATTAACG-3´ were used as in the first step. For sequencing, the inner primers and the primers CpsF2 5´-YGTAGGTGCACGYGGAG-3´ and 201FAG 5´-GGAGCIGARTTCCAATACGCTCAITC-3´ were used together with a BigDye terminator labeled sequencing kit (Applied Biosystems (Foster City, CA, USA).

We found that 10% of 431 juvenile fulmars were infected with *Chlamydophila* spp. Detection rates ranged from 7% to 21% in different collection areas. Since the juvenile birds were caught on the sea surface near their nesting cliffs before they were fledged, the different detection rates suggest that the prevalence of infection varies among colonies. This hypothesis is supported by the fact that ringing of fulmars shows their lifestyle is local, that is, they do not travel over long distances. The reported *C. psittaci* prevalence in other studies of wild birds varies widely, from 0% to 74% (*6**,**7*). This wide range is partly attributable to small and selected study populations and to the use of insensitive techniques or methods that measure exposure rather than prevalence.

Adult birds often have nonsymptomatic infections, while young birds frequently have acute disease. These descriptions are consistent with those from epidemics in the Faroes, where humans contracted chlamydophilosis only when handling juvenile fulmars, not when catching adult birds (*3*).

Analysis of *ompA* from 29 representative specimens from the *Chlamydophila*-positive birds showed no variation and showed sequences almost identical (T471C mutation in the variable domain 2, GenBank accession no. AM050561) to that of the prototype strain 6BC for *C. psittaci*. This finding is in contrast to our previous study of seabirds, in which we found a *C. psittaci* strain closely related to *C. abortus* (*8**,**9*). To our knowledge, further sequence data on *Chlamydophila* infections in seabirds have not been reported.

## Conclusions

Our finding of a detectable infection rate of 10% is representative of juvenile fulmars; however, in adult fulmars the rate is probably lower, since they may develop immunity and are less exposed to dust contaminated by *C. psittaci* in nests. Serotyping and genotyping of *C. psittaci* strains are based on the major outer membrane protein coded by *ompA*, and at least 6 types are described. Type A is considered to have psittacine birds as natural hosts, and types B and D are associated with pigeons and turkeys, respectively; the natural hosts for types C, E, and F are unknown. The *ompA* sequence in infected fulmars is almost identical to the 6BC isolate, a strain isolated 64 years ago in a parakeet (*10*). Knowledge of the correlation between *C. psittaci* types and wild bird species as natural hosts, however, is limited. The question is further complicated by the fact that birds are excellent vectors and may contract *C. psittaci* when they feed on the detritus of infected animals of all kinds. Our *ompA* data may support the speculation that fulmars in the Faroes acquired *C. psittaci* from infected and dead parrots thrown overboard during shipment from Argentina to Europe in 1930 (*11*). The first human case in the Faroes appeared that year on the southernmost island of Suduroy, and during the years 1933–1938 severe outbreaks occurred on Sandoy and other islands (Figure) (*3*). In Iceland, the first human chlamydophilosis cases linked to fulmars were reported on the Vestmanna Islands in 1939. Six cases were reported; all occurred after birds had been prepared for human consumption (H. Briem, pers. comm.) This finding further supports the hypothesis that *C. psittaci* was spread from ships by psittacine birds to fulmars in the northern Atlantic and then was gradually introduced to more distant areas.

Catching young fulmars in the Faroes was prohibited in 1938. Only sporadic episodes of chlamydophilosis were observed after the new legislation. Since 1954, when the ban on taking fulmars was lifted, and until 2003, the chief medical officer has reported 48 cases. Apart from an outbreak of 8 cases in 1972, the annual number of cases has ranged from 0 to 3, none fatal. On average, 2.2 cases per 100,000 inhabitants have been reported, which is much higher than figures from other countries where chlamydophilosis is a reportable disease. Also, awareness of chlamydophilosis is probably considerably higher in the Faroes than in the other countries, a fact that may lead to higher detection rates. What proportion of human chlamydophilosis cases in the Faroes is associated with fulmars is unknown, but since 50,000–100,000 juvenile fulmars are prepared for human consumption each year, and *C. psittaci* prevalence is 10%, up to 10,000 potential human exposures to *C. psittaci* occur yearly. The risk of humans acquiring symptomatic *C. psittaci* infection from fulmars is thus very low. This conclusion agrees with our previous finding that bird ringers were antibody-negative for *C. psittaci*, despite high exposure to birds (*12*). A contrasting report of a human chlamydophilosis outbreak associated with wild birds in Australia has recently been published. Clinical, laboratory and epidemiologic data indicate disease episodes related to bird contact and lawn mowing, but the source of infection could not be identified (*13*).

Nevertheless, unanswered questions remain. Why have more chlamydophilosis cases not been noted after the taking of fulmars was resumed in 1954? Why did the annual incidence decrease from 42 per 100,000 inhabitants during the epidemic period in the 1930s to 2.2 after 1954? If a *C. psittaci* infection was introduced among fulmars in 1930 as a result of shipping parrots to Europe, an initially high attack rate could have been expected. Subsequently, adaptation between bacteria and host may have led to less symptomatic infections and lower shedding of bacteria. The ensuing decrease in human exposure to *C. psittaci* may have resulted in a falling incidence of disease. An alternative explanation for the low incidence of chlamydophilosis in recent years may be a general improvement in public health. By analogy with the eye disease trachoma, caused by *Chlamydia trachomatis*, the incidence of chlamydophiliosis would be expected to decrease as hygienic conditions improved. However, human chlamydophilosis is a zoonosis, and the impact of improved public health is not clear. A third explanation for the lower disease rates could be changes in the methods used to prepare caught fulmars. No systematic investigation has been made of changes in handling procedures, and identifying any specific modification that might have led to major reductions in exposure to infected birds is difficult. Thus, this issue is still unresolved.

*C. psittaci* is rarely an agent of community-acquired pneumonia (*14*), and in outbreaks related to pet birds the disease may be mild rather than severe (*15*). Consequently, investigation for chlamydophilosis in humans should only be considered when clinical and epidemiologic data indicate such a diagnosis.
